# Bone Progenitors Pull the Strings on the Early Metabolic Rewiring Occurring in Prostate Cancer Cells

**DOI:** 10.3390/cancers14092083

**Published:** 2022-04-21

**Authors:** Pablo Sanchis, Nicolas Anselmino, Sofia Lage-Vickers, Agustina Sabater, Rosario Lavignolle, Estefania Labanca, Peter D. A. Shepherd, Juan Bizzotto, Ayelen Toro, Antonina Mitrofanova, Maria Pia Valacco, Nora Navone, Elba Vazquez, Javier Cotignola, Geraldine Gueron

**Affiliations:** 1 Laboratorio de Inflamación y Cáncer, Departamento de Química Biológica, Facultad de Ciencias Exactas y Naturales, Universidad de Buenos Aires, Buenos Aires C1428EGA, Argentina; pablosanchis@qb.fcen.uba.ar (P.S.); sofilage@qb.fcen.uba.ar (S.L.-V.); asabater@qb.fcen.uba.ar (A.S.); rlavignolle@qb.fcen.uba.ar (R.L.); jbizzotto@qb.fcen.uba.ar (J.B.); ayelentoro@qb.fcen.uba.ar (A.T.); pvalacco@qb.fcen.uba.ar (M.P.V.); elba@qb.fcen.uba.ar (E.V.); jcotignola@qb.fcen.uba.ar (J.C.); 2 CONICET-Universidad de Buenos Aires, Instituto de Química Biológica de la Facultad de Ciencias Exactas y Naturales (IQUIBICEN), Buenos Aires C1428EGA, Argentina; 3 Department of Genitourinary Medical Oncology and The David H. Koch Center for Applied Research of Genitourinary Cancers, The University of Texas MD Anderson Cancer Center, Houston, TX 77030, USA; nanselmino@mdanderson.org (N.A.); elabanca@mdanderson.org (E.L.); pshepherd@mdanderson.org (P.D.A.S.); nnavone@mdanderson.org (N.N.); 4 Universidad Argentina de la Empresa (UADE), Instituto de Tecnología (INTEC), Buenos Aires C1073AAO, Argentina; 5 Department of Biomedical and Health Informatics, Rutgers School of Health Professions, Rutgers Cancer Institute of New Jersey, New Brunswick, NJ 07101, USA; amitrofa@shp.rutgers.edu

**Keywords:** prostate cancer, bone metastasis, metabolism, gene signature, lipid metabolism, PKA

## Abstract

**Simple Summary:**

Almost 90% of prostate cancer metastases occur in bone. The arrival of tumor cells to the homing organ requires a metabolic adaptation to different nutrients and oxygen availability in the new microenvironment to fulfill sustained growth and proliferation rates. In this study, we characterized the alterations in tumor metabolism occurring in human prostate cancer cells when they interact with bone cells. We hypothesized that elucidating how these cells obtain their energy and “building blocks”, and that identifying key determinants underlying this phenomenon, could be a promising strategy to halt disease progression. Accordingly, we discovered five genes related to the metabolism of lipids that play a critical role in the survival of metastatic patients, and we established a communication axis between tumor and bone cells that includes bone-secreted collagen and the tumor Protein Kinase A, which drives the early metabolic reprogramming of metastatic cells.

**Abstract:**

Metastatic prostate cancer (PCa) cells soiling in the bone require a metabolic adaptation. Here, we identified the metabolic genes fueling the seeding of PCa in the bone niche. Using a transwell co-culture system of PCa (PC3) and bone progenitor cells (MC3T3 or Raw264.7), we assessed the transcriptome of PC3 cells modulated by soluble factors released from bone precursors. In a Principal Component Analysis using transcriptomic data from human PCa samples (GSE74685), the altered metabolic genes found in vitro were able to stratify PCa patients in two defined groups: primary PCa and bone metastasis, confirmed by an unsupervised clustering analysis. Thus, the early transcriptional metabolic profile triggered in the in vitro model has a clinical correlate in human bone metastatic samples. Further, the expression levels of five metabolic genes (*VDR, PPARA, SLC16A1, GPX1* and *PAPSS2*) were independent risk-predictors of death in the SU2C-PCF dataset and a risk score model built using this lipid-associated signature was able to discriminate a subgroup of bone metastatic PCa patients with a 23-fold higher risk of death. This signature was validated in a PDX pre-clinical model when comparing MDA-PCa-183 growing intrafemorally vs. subcutaneously, and appears to be under the regulatory control of the Protein Kinase A (PKA) signaling pathway. Secretome analyses of conditioned media showcased fibronectin and type-1 collagen as critical bone-secreted factors that could regulate tumoral PKA. Overall, we identified a novel lipid gene signature, driving PCa aggressive metastatic disease pointing to PKA as a potential hub to halt progression.

## 1. Introduction

No curative therapy is currently available for metastatic PCa. Late-stage treatment options include hormonal therapy (HT), chemotherapy, and/or radiation therapy [1]. Approaches targeting aberrantly activated pathways in advanced PCa are being applied in the clinic, including PI3K pathway blockade, inhibitors of DNA damage response (such as PARP inhibitors), and prostate-specific membrane antigen (PSMA) targeting [2,3]. An alternative option for some men with advanced PCa that no longer responds to hormones is a cancer vaccine tailored to trigger the patient’s immune system to specifically attack PCa cells [1], but its effectiveness remains limited. Despite these advances, treatment responses are heterogeneous, and, in many cases, the disease develops resistance, stressing the need for a better understanding of PCa biology to improve patient selection strategies.

Among metastatic prostate cancer (PCa), the incidence of bone metastasis reaches 88% [4] and represents a severe clinical hurdle to overcome. Bidirectional interactions between bone and PCa cells suggest that not only tumor-cell-derived factors affect bone cell physiology, but also the bone cells can stimulate metastatic prostate tumor growth. The arrival of tumor cells to the target homing organ requires a metabolic adaptation to different nutrients and oxygen availability in the new microenvironment [5], which could be mediated by bone-secreted factors. The identification of factors in the bone microenvironment that promote tumor metabolic rewiring would be an important step in delineating the adaptation mechanisms and PCa bone progression.

Up to date, adipocytes have been one of the cell types from the bone marrow described as promoters of the metabolic reprogramming occurring in PCa cells [6,7]. Bone metastatic PCa cells uptake fatty acids (FA) released from adipocytes through the fatty acid-binding protein 4 (FABP4) [8]. Adipocyte-derived lipids may contribute to a switch towards aerobic glycolysis in tumor cells, dependent on HIF-1α activation [9]. Excessive lactate production by aerobic glycolysis and the resulting low extracellular pH has been associated with the activation of metalloproteases, promoting cancer invasion and metastasis [10].

Interestingly, PCa progression has been associated with an increase in de novo FA synthesis independently from the systemic lipid levels [11], underlined by an overexpression of lipogenic enzymes, including FA synthase (FASN) [12,13], which correlates with biochemical relapse of PCa patients, independently from Gleason score [14]. Inhibition of FASN with an irreversible inhibitor (IPI-9119) has led to a decreased castration-resistant PCa (CRPC) growth in vivo [15]. However, it has been reported that the response to FASN inhibitors cannot be predicted by FASN expression levels [16], imposing a difficulty in the selection of candidate patients for whom these therapies might be promising.

Thus, the discovery of novel metabolic gene expression patterns/signatures and processes that orchestrate PCa progression in the bone might be a promising approach for the identification of novel therapeutic druggable targets to prevent metastatic progression.

In this work, we used a co-culture transwell system to identify key metabolic gene alterations associated with the crosstalk between PCa and bone progenitor cells (pre-osteoblasts or pre-osteoclasts). We performed an extensive bioinformatics analysis in human metastatic PCa samples and have demonstrated that an unsupervised clustering using the gene signature found in vitro successfully distinguished primary from metastatic human PCa samples. Further, we obtained secretome profiles from co-cultured bone progenitors with PCa cells, and established a plausible molecular communication axis by which bone cells lure tumor cells towards the bone niche.

## 2. Materials and Methods

### 2.1. Cell Culture

#### 2.1.1. Cell Lines

Human PC3 (metastatic prostate cancer cell line) cells were obtained from the American Type Culture Collection (ATCC, Manassas, VA, USA) and were routinely cultured using RPMI 1640 (Invitrogen, Grand Island, NY, USA) supplemented with 10% fetal bovine serum (FBS) (Internegocios, Mercedes, Buenos Aires, Argentina). MC3T3 (pre-osteoblastic cell line) cells were cultured in α-MEM (Invitrogen, Grand Island, NY, USA) supplemented with 10% FBS. Raw264.7 (pre-osteoclastic cell line) cells were cultured in DMEM supplemented with 5% FBS.

#### 2.1.2. Co-Culture System

PC3 cells were co-cultured with MC3T3 or Raw264.7 cells using an in vitro bio-compartment culture system to generate a model of PCa bone metastases as previously described [17]. PC3 cells were seeded at a density of 100,000 cells/insert in 6-well plate cell-culture inserts (0.4 mm pore; Falcon/Becton Dickinson Labware, Franklin Lakes, NJ, USA). The osteoblast/osteoclast precursor cells were seeded in 6-well culture plates at a density of 200,000 and 300,000 cells/well, respectively. After 24 h, inserts containing PC3 cells were washed three times with PBS and placed into tissue-culture plates containing the MC3T3 or Raw264.7 cells. In this indirect co-culture system, the two different cell lines shared the culture medium but were not in physical contact. Co-culturing of PC3 cells with pre-osteoblasts (MC3T3) or pre-osteoclasts (Raw264.7) was performed with α-MEM or DMEM, respectively, supplemented with 2% FBS for 24 h and cells were harvested separately. As control, each cell line (PC3, MC3T3 and Raw264.7) was grown alone using the same culture conditions described above [17,18].

### 2.2. RNA Isolation and Sequencing

Total RNA of each cell line in each condition was isolated using Quick-Zol (Kalium technologies, Bernal, Buenos Aires, Argentina) according to the manufacturer’s protocol. The sequencing libraries for the RNA samples were prepared as previously described [16]. One replicate for each condition consisting of the RNA pooled from 5 independent experiments was used for the RNA-seq.

### 2.3. Bioinformatics Analyses

#### 2.3.1. Differential Expression Analysis and Reactome Pathway Database

After mapping RNA-seq reads to the human GRCh38 reference genome using HISAT2 [19], differential expression analysis was performed using the GFOLD algorithm (c = 0.01) [20], for PC3 cells co-cultured with MC3T3 or Raw264.7 cells compared with PC3 alone. The calculated GFOLD can be interpreted as a reliable log2 fold change, where GFOLD > 0 means over-expression, GFOLD < 0 means under-expression and GFOLD = 0 represents unchanged gene expression levels. After performing the differential expression analysis, we based our work on the Reactome classification [21] to obtain the differentially expressed genes (DEGs) associated to the category “Metabolism”.

#### 2.3.2. Pathway Enrichment Analysis

The metabolic genes identified in the Reactome pathway database were subjected to Gene set enrichment analysis (GSEA) (Broad Institute) (10,000 permutations were run), using the clusterProfiler [22] and enrichplot [23] R packages, using the KEGG pathway collection [24] and the Gene Ontology (GO) database [25].

Ingenuity Pathway Analysis (IPA, QIAGEN Inc., Germantown, MA, USA) [26] was used to study upstream regulators. Statistical significance was set at *p* < 0.05.

#### 2.3.3. Metastatic PCa Patient Cohorts

To compare gene expression profiles in metastatic samples vs. primary tumors, we used the GSE74685 dataset from Fred Hutchinson Cancer Research Center, which comprises 171 samples from primary prostatic (n = 14) or metastatic (n = 149) tumors from 63 PCa patients, with expression data obtained from complete Agilent 44K whole human genome expression oligonucleotide microarray [27].

Moreover, the dataset from the SU2C-PCF Dream Team: Precision Therapy for Advanced Prostate Cancer comprising whole-exome sequencing of 444 CRPC tumors was analyzed. It contains clinical, transcriptomic and survival information for 70 metastatic samples from CRPC tumors [28].

Supplementary Table S1 shows the Gene Name, Gene Symbol, Entrez Gene ID and Species for every gene of interest assessed in this work.

#### 2.3.4. Principal Component Analysis (PCA)

This dimensionality reduction algorithm was performed using factoextra package in R [29]. Unsupervised clustering analysis including expression data of the dysregulated metabolic genes was performed using ggplot2 [30] and pheatmap [31] packages in R.

#### 2.3.5. Risk Scoring System Analysis

Based on gene expression, a risk score model was created using the coefficients of a multivariable Cox logistic-regression analysis for metastatic patients. The patient risk score was calculated as the sum of the product of Cox coefficients (Coef) values of all genes and the dichotomized (low/high) expression (Expr) of all genes: risk score = $\sum_{i=1}^{n}\left(Coef_{i}\times Expr_{i}\right)$. We used the Cutoff Finder software [32] to categorize patients into high-risk score and low-risk score groups. KM survival analysis was used to determine whether OS was significantly different between high-risk and low-risk patients.

### 2.4. Secretome Analysis of Conditioned Media

In-depth proteomic analysis LC ESI-MS/MS was performed using the conditioned media (CM) obtained from the co-culture experiment of PC3 cells with Raw264.7 or MC3T3 cells as previously described [17]. The obtained MS/MS spectra were compared against human and murine protein databases to identify proteins in each sample. We then compared the proteins in each condition, to obtain a list of secreted proteins in the co-culture of PC3 with bone progenitors. PKA–secretome interactions were evaluated using STRING (https://string-db.org/, accessed on 13 October 2021).

### 2.5. PKA Inhibition and ATP Content Measurement

PC3 cells were seeded at a density of 80,000 cells/well in a 12-multiwell cell-culture plate. After 24 h, culture media was replaced by 500 µL of CM from PC3 grown alone or in co-culture with MC3T3 for 24 h, 10 µM of H-89 dihydrochloride hydrate (H89, a PKA inhibitor [33]) (Sigma, Burlington, MA, USA) or vehicle was added to the media (3 h). ATP content was measured using the CellTiter-Glo Luminescent Cell Viability Assay (Promega, Madison, WI, USA) following manufacturer’s instructions, and relativized to cell number.

### 2.6. RT-qPCR

cDNAs were synthesized with RevertAid Premium First Strand cDNA Synthesis Kit (Fermentas, Waltham, MA, USA) and used for real-time PCR amplification with Taq DNA Polymerase (Invitrogen, Waltham, MA, USA) in a QuantStudio 3 Real-Time PCR System (Thermo Fisher Scientific, Waltham, MA, USA), as previously described [17]. PPIA was used as the internal reference gene. Data obtained were analyzed using the method of 2^−ΔΔCT^ [34]. Primers sequences for each gene are shown in Supplementary Table S2.

### 2.7. Animals

All practices involving laboratory animals were approved by the Institutional Animal Care and Use Committee of The University of Texas MD Anderson Cancer Center, under the regulation of the Animal Welfare Committee (IACUC) and conform to the NIH Policy on Human Care and Use of Laboratory Animals.

### 2.8. MDA-PCa-183 Patient-Derived Xenograft (PDX) Generation and RNA Sequencing

The PDX MDA-PCa-183 was developed in the Laboratory of Dr. Navone at the “Prostate Cancer Patient Derived Xenografts Program” at the MD Anderson Cancer Center and the David H. Koch Center for Applied Research of Genitourinary Cancers, from a bone marrow aspirate of a human male with metastatic adenocarcinoma as described previously [35]. Briefly, tissue derived MDA-PCa-183 PDX was injected either subcutaneously (*s.c.*) or intrafemorally (IF) into the distal end of right femurs of 6- to 8-wk-old male CB17 *SCID* mice according to published protocols [36,37]. Left legs were sham-injected non-tumor-bearing controls. Tumors’ RNA was extracted, purified, and sequenced (RNA-seq) as previously described [38].

### 2.9. Statistical Analysis

Wilcoxon or Kruskal–Wallis tests were used for testing differences in gene expression across tissue samples. Log-rank test and Cox proportional hazard model regression were employed to assess the significance of gene expression on patients’ survival. Multivariable analyses were performed in Stata Software and plotted in GraphPad Prism software (La Jolla, CA, USA).

Kaplan–Meier (KM) curves showing overall survival (OS) of patients with metastases were plotted using the survminer package [39] in R. To find the optimal cutoff value to stratify patients into two groups based on the expression levels, we used the Cutoff Finder tool. Statistical significance was set at *p* < 0.05.

## 3. Results

### 3.1. Differential Transcriptomic Analysis of PC3 and Bone Cells Growing in a Co-Culture Transwell System

**Figure 1:**
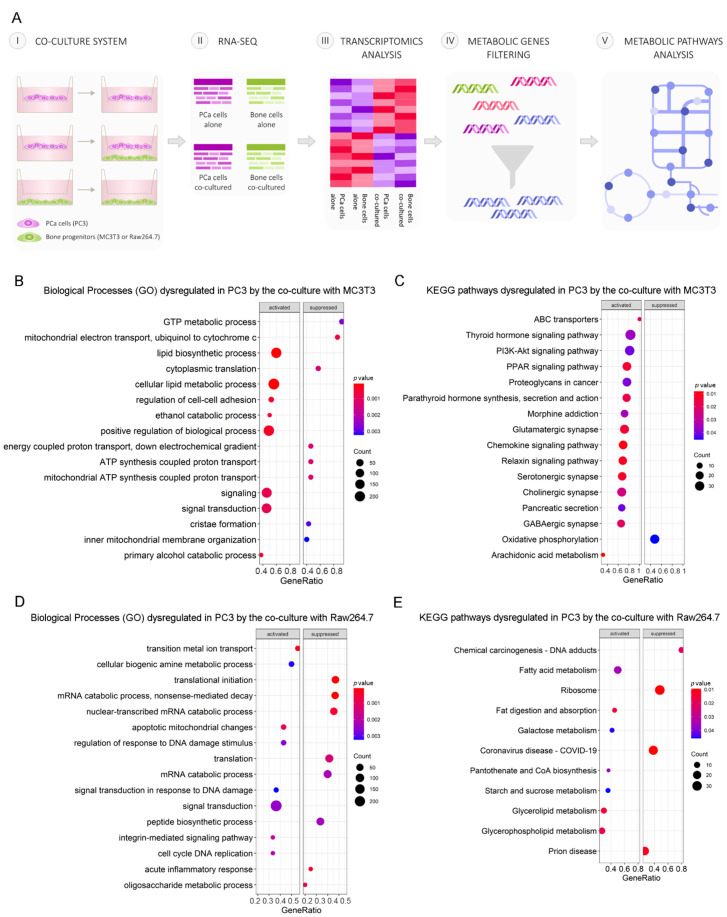
Schematic representation of the indirect co-culture system and alterations of tumor cells’ metabolic profile induced by the co-culture with bone progenitors. (**A**) PC3 cells were cultured alone or co-cultured with bone progenitors (MC3T3 or Raw264.7) using an in vitro bio-compartment, which allows cells to share the culture medium and signaling factors without physical contact, in order to mimic the interactions between tumor cells and the bone metastatic niche through soluble factors. Cells were seeded in their respective compartments and after 24 h the inserts containing PC3 cells were washed and placed in the co-culture plates with or without osteoblast/osteoclast precursor cells. On day 3, PC3 cells were harvested, RNA was extracted and an RNA-seq with differential expression analysis was performed. The DEG’s associated with metabolism categories were identified using Reactome. (**B**–**D**) Enrichment plots (GSEA) showing the significantly activated and repressed categories after the co-culture of PC3 cells with either osteoblast or osteoclast precursor cells. (**B**) Gene ontology (GO) biological processes categories and (**C**) KEGG pathways significantly dysregulated in PC3 cells after the co-culture with MC3T3 cells. (**D**) GO biological processes categories and (**E**) KEGG pathways significantly dysregulated in PC3 cells after the co-culture with Raw264.7 cells. The size of the dots represents the number of counts observed for each category, and the color gradient shows the *p* value. Statistical significance was set at *p* < 0.05.

PCa progression is driven by communication between prostate and bone cells in the metastatic niche. To study how cell–cell communication induces metabolic alterations at the transcriptional level in PCa cells, we performed RNA-seq on PC3 cells co-cultured with osteoblastic (MC3T3) or osteoclastic (Raw264.7) bone progenitors, followed by a gene expression analysis, comparing co-cultured PC3 cells vs. PC3 alone (control) (Figure 1A). After differential gene expression analyses, we focused on identifying dysregulated genes associated with the “Metabolism” category, using the Reactome pathway database [21]. To delineate the metabolic-transcriptomic phenotype of co-cultured PCa cells with bone cells, a Gene Set Enrichment Analysis (GSEA) was performed to identify Gene Ontology (GO) biological processes and KEGG pathways involved in this model. We found that PC3 cells co-cultured with pre-osteoblastic MC3T3 cells presented an activation of lipid metabolism (lipid biosynthetic process, cellular lipid metabolic process), while genes related to the electron transport chain (mitochondrial electron transport ubiquinol to cytochrome c, mitochondrial ATP synthesis coupled proton transport) were suppressed in comparison to PC3 cells cultured alone (Figure 1B). The KEGG pathways analysis revealed an upregulation of several metabolic regulatory pathways, including PPAR and PI3K-Akt signaling pathways, and a downregulation of the oxidative phosphorylation pathway (Figure 1C). When PC3 cells were co-cultured with osteoclastic progenitors Raw264.7, cell-cycle-related categories (signal transduction in response to DNA damage, cell cycle DNA replication) were activated, and the ones related to protein processing (translational initiation, peptide biosynthetic pathway) and metabolism of RNA (mRNA catabolic process, nonsense-mediated decay, nuclear-transcribed mRNA catabolic process) were suppressed (Figure 1D). Among the enriched KEGG pathways, lipid metabolism-related categories appeared as the most significantly activated (Figure 1E).

These results evidence a strong dysregulation of lipid-related metabolic pathways in PCa cells induced by the indirect interaction with bone cells.

### 3.2. Clinical Correlation of Metabolic Genes Dysregulated in PC3 Co-Cultured with Bone Progenitors and Human PCa Metastatic Samples

**Figure 2:**
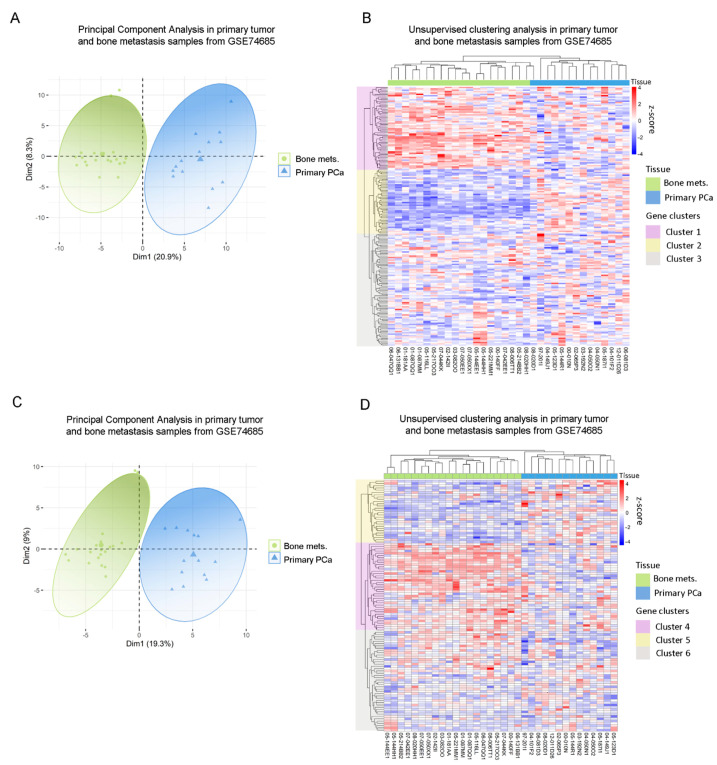
Clinical correlation of metabolic genes dysregulated in PC3 by the co-culture with bone progenitors. (**A**) Principal Component Analysis (PCA) biplot considering the expression of the metabolic genes identified in PC3 cells co-cultured with MC3T3 showing a rough segregation of primary tumors (blue) and bone metastases samples (green). Each point represents one sample from the GSE74685 dataset. (**B**) Heatmap depicting an unsupervised clustering analysis in human primary tumor and bone metastases samples, considering the expression of metabolic genes dysregulated in PC3 cells co-cultured with MC3T3 cells. (**C**) PCA biplot considering the expression of the metabolic genes identified in PC3 cells co-cultured with Raw264.7 showing a rough segregation of primary tumors (blue) and bone metastases samples (green). Each point represents one sample from the GSE74685 dataset. (**D**) Heatmap depicting an unsupervised clustering analysis in human primary tumor and bone metastases samples, considering the expression of metabolic genes dysregulated in PC3 cells co-cultured with Raw264.7 cells. Red, white and blue colors represent higher, equal or lower z-score expression levels, respectively. Each green or blue box at the top of the heatmap represents a bone metastasis or primary tumor sample, respectively. Purple, yellow and gray boxes on the left side of the heatmap enclose genes classified in Cluster 1, 2 and 3, or 4, 5 and 6, respectively. Statistical significance was set at *p* < 0.05.

Next, we sought to assess whether gene expressions from the previously identified dysregulated metabolic pathways observed in our in vitro model had a correlate in human PCa metastatic samples from the GSE74685 dataset [27]. Indeed, the metabolic differentially expressed genes (DEGs) between PC3 cells co-cultured with MC3T3 compared with the control efficiently discriminated patient samples in a PCA according to their tissue of origin: primary PCa or PCa bone metastasis (PC1 = 20.9%) (Figure 2A) This grouping was also confirmed in an unsupervised clustering analysis (Figure 2B). Additionally, the clustering defined three gene clusters: genes with high expression in bone metastases and low expression in primary tumors (Cluster 1); genes with low expression in bone metastatic samples and high expression in primary tumors (Cluster 2); and genes without a clear/distinct expression profile in both tissues (Cluster 3) (Figure 2B).

GO analyses for Cluster 1 revealed a significant enrichment of genes associated with lipid and fatty acid (FA) metabolism (Supplementary Figure S1A), while for Cluster 2 the most significant categories included nucleic acid metabolism, sulfuration and the regulation of biosynthetic processes (Supplementary Figure S1B).

In parallel, the same analysis was performed with the metabolic DEGs obtained from the PC3/Raw264.7 co-culture. Accordingly, two clearly defined sample groups were identified in the PCA, corresponding to primary prostate tumors and bone metastases (PC1 = 19.3%) (Figure 2C). When performing the unsupervised hierarchical clustering analysis, similar results were observed for this gene list, which was able to accurately discriminate samples according to their tissue of origin (Figure 2D). There were also three gene clusters, each of them with a distinctive expression pattern: genes with high expression in bone metastases and low expression in primary tumors (Cluster 4); genes with low expression in bone metastases and high expression in primary tumors (Cluster 5); and genes without a clear expression profile (Cluster 6) (Figure 2D). Interestingly, Cluster 4 was associated to ion transport and macromolecular complex organization, among others (Supplementary Figure S1C), while Cluster 5 was mainly associated with carbohydrate-related pathways (gluconeogenesis, hexose, fructose, and pyruvate metabolism) and FA metabolism (Supplementary Figure S1D).

To further validate these results and ensure that they are not random, we performed the unsupervised hierarchical clustering analysis using 200 randomly selected genes, which did not discriminate samples according to their precedence (Supplementary Figure S1E).

These results show that the expression of the DEGs identified in the co-culture system was able to discriminate clinical metastases from primary PCa samples and point out to the clinical relevance of the metabolic reprogramming occurring during disease progression.

### 3.3. Analysis of the Metabolic-Related Gene Expression Profile Associated with Overall Survival in Human Metastatic PCa

**Figure 3:**
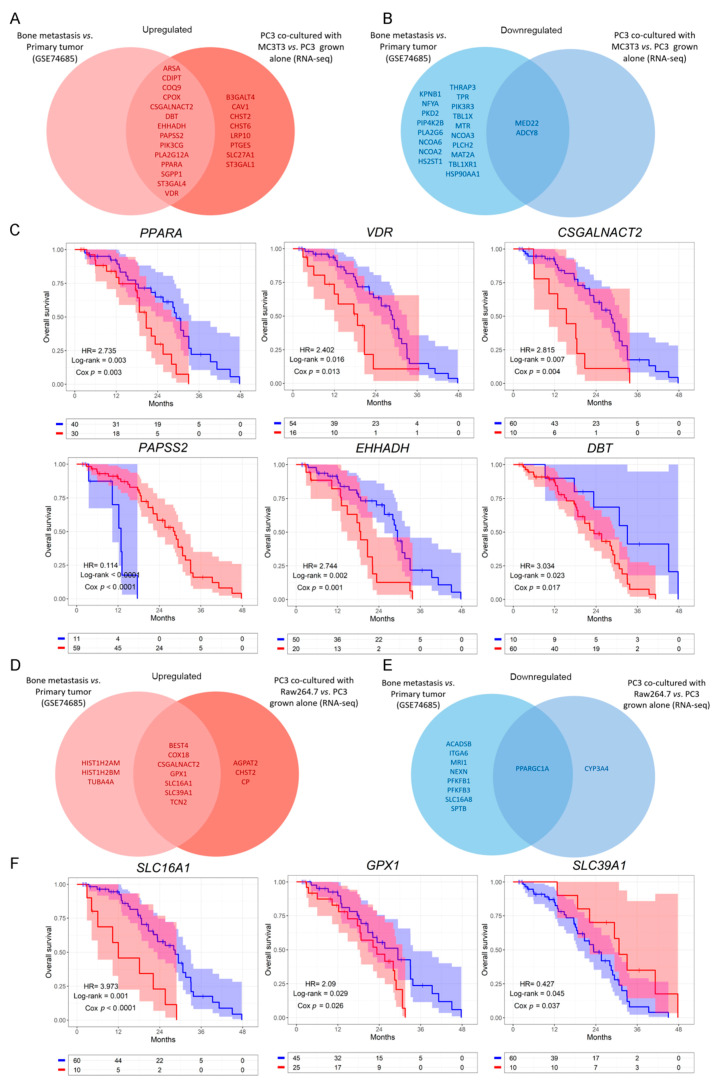
Dysregulated metabolic-related genes in co-cultured PC3 cells compared to PC3 grown alone, and in metastatic compared to primary PCa samples. (**A**,**B**) Venn diagram showing the upregulated (**A**) or downregulated (**B**) genes obtained from a differential expression analysis performed on patients’ bone metastases vs. primary tumor samples (GSE74685), and the significantly upregulated/downregulated genes in PC3 co-cultured with MC3T3 cells. (**C**) Kaplan–Meier (KM) curves using the SU2C-PCF dataset for overall survival (OS) of PCa patients segregated based on the expression levels of the clinically relevant metabolic-associated genes identified in both the co-culture system with MC3T3 cells and the GSE74685 dataset. (**D**,**E**) Venn diagram showing the upregulated (**D**) or downregulated (**E**) genes obtained from a differential expression analysis performed on patients’ bone metastases vs. primary tumor samples (GSE74685), and the significantly up/down regulated genes in PC3 co-cultured with Raw264.7 cells. (**F**) KM curves using the SU2C-PCF dataset for OS of PCa patients segregated based on the expression levels of the clinically relevant metabolic-associated genes identified in both, the co-culture system with Raw264.7 cells and the GSE74685 dataset. The optimal cutoff value for gene expression was determined using the Cutoff Finder tool. All comparisons consider low expression patients as the reference group. OS of patients with high (red) vs. low (blue) expression for each gene. HR = hazard ratios [95% confidence interval]. All comparisons consider low expression patients as the reference group. Cox *p* = Cox proportional hazard model *p* value. Statistical significance was set at *p* < 0.05.

Next, we evaluated whether the genes that were dysregulated in the in vitro model were also dysregulated in the same way when comparing bone metastases with primary PCa samples from the GSE74685 dataset [27]. Among the genes comprised in the GO categories significantly represented in Cluster 1, 14 were upregulated in both the PC3/MC3T3 co-culture and the human bone metastases, while 8 genes were only upregulated in the co-culture system (Figure 3A and Supplementary Figure S2). Regarding Cluster 2, only two genes were downregulated in both conditions (Figure 3B, right panel and Supplementary Figure S2).

To further study the clinical implications of our findings in metastatic PCa patients, we explored the SU2C-PCF dataset [28], which contains transcriptomic, clinical and overall survival (OS) data. We evaluated patient survival associated with the expression of the genes selected in Figure 3A,B. The results show that high expression of *CSGALNACT2*, *VDR*, *PPARA*, *DBT* and *EHHADH* was significantly associated with a worse OS (HR = 2.815, Cox *p* = 0.004, for *CSGALNACT2*; HR = 2.402, Cox *p* = 0.013, for *VDR*; HR = 2.735, Cox *p* = 0.003, for *PPARA*; HR = 3.034, Cox *p* = 0.017, for *DBT*; HR = 2.744, Cox *p* = 0.001, for *EHHADH*) (Figure 3C), while high expression of *PAPSS2* was associated with a better prognosis in metastatic PCa patients (HR = 0.114, Cox *p* < 0.0001) (Figure 3C). No significant differences were observed for the remaining genes (Supplementary Figure S3A).

Further, from the genes identified in the PC3/Raw264.7 co-culture, seven were upregulated (Figure 3D and Supplementary Figure S4) and only one was downregulated in both conditions (Figure 3E and Supplementary Figure S4). *CSGALNACT2* was previously shown to be associated with a shorter OS.

Interestingly, high expression of *GPX1* and *SLC16A1* was associated with a poor OS for metastatic PCa patients from the SU2C-PCF dataset (HR = 2.09, Cox *p* = 0.029, for *GPX1*; HR = 3.973, Cox *p* < 0.0001, for *SLC16A1*) (Figure 3F), while high expression of *SLC39A1* correlated with a better prognosis (HR = 0.427, Cox *p* = 0.037) (Figure 3F). No significant effects on the OS were observed for the remaining genes (Supplementary Figure S3B).

These results reflect that the metabolic transcriptomic profile dysregulated in the in vitro model recapitulates what is observed in bone metastases samples (Figure 2 and Figure 3). Moreover, survival analyses evidenced an association between altered transcriptomics of these genes and the OS, highlighting their clinical relevance in metastatic PCa.

### 3.4. Defining a Metabolic Gene Signature Associated with Metastatic PCa

**Figure 4:**
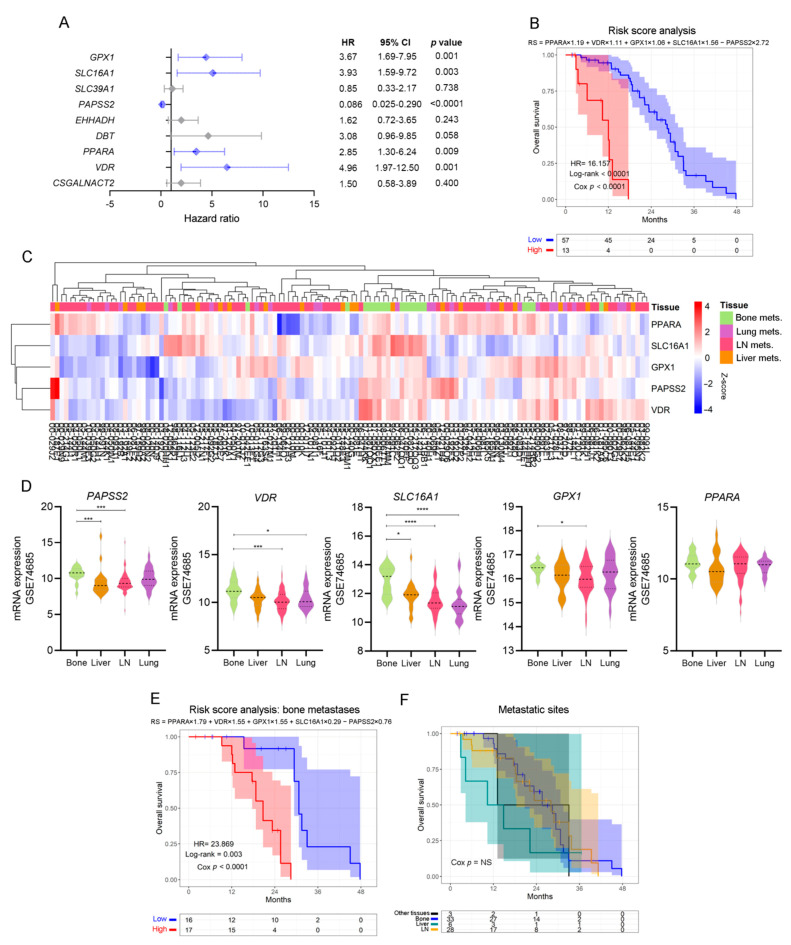
Patient stratification by independent, lipid metabolic-associated death risk predictors. (**A**) Multivariable analyses presented by a forest plot between statistically significant genes in the univariable analyses, using metastatic PCa patients’ data from the SU2C-PCF dataset. All comparisons consider low expression patients as the reference group. Light blue lines correspond to statistically significant independent genes (*p* < 0.05). (**B**) KM curve for OS in high-risk (red) and low-risk (blue) groups, according to a risk score model based on the expression of *PPARA*, *VDR, SLC16A1, GPX1* and *PAPSS2*, in PCa metastatic patients from the SU2C-PCF dataset. (**C**) Heatmap depicting an unsupervised clustering analysis showcasing the expression of metastatic samples from the GSE74685 dataset, considering the expression of the metabolism-associated risk signature. Red, white and blue represent higher, equal or lower z-score expression levels, respectively. Kruskal–Wallis test was used to assess differential gene expression. (**D**) Violin plots depicting *PAPSS2*, *VDR*, *SLC16A1*, *GPX1*, and *PPARA* expression levels in metastatic samples from the GSE74685 dataset. (**E**) KM curve for OS in high-risk (red) and low-risk (blue) groups, according to a risk score model based on the expression of the gene signature in bone metastases from the SU2C-PCF dataset. (**F**) KM curve for OS of metastatic patients segregated based on the metastatic site from the SU2C-PCF dataset. Yellow curve: LN metastases; green curve: liver metastases; blue curve: bone metastases; black curve: other metastases. LN: lymph node. Mets: metastasis. Statistical significance was set at *p* < 0.05. * *p* < 0.05, *** *p* < 0.001, **** *p* < 0.0001. HR = hazard ratio [95% confidence interval].

In light of the results previously obtained, we sought to validate the potential of these biological markers as independent risk predictors in PCa. We performed a multivariable Cox proportional hazards analysis including the genes that significantly altered OS in the univariable analyses (Figure 3). Interestingly, *VDR* (HR = 4.96, Cox *p* = 0.001)*, PPARA* (HR = 2.85, Cox *p =* 0.009), *SLC16A1* (HR = 3.93, Cox *p* = 0.003), *GPX1* (HR = 3.67, Cox *p* = 0.001) and *PAPSS2* (HR = 0.086, Cox *p* < 0.0001) were independent risk factors (Figure 4A), which have been associated with the lipid metabolism.

We next performed a multivariable Cox regression analysis and built a prognostic model for predicting OS as follows: $\sum_{i=1}^{n}\left(Coef_{i}\times Expr_{i}\right)\;$(Supplementary Table S3A).

Accordingly, patients with high-risk scores had worse clinical outcomes than patients with low-risk scores (HR = 16.16, Cox *p* < 0.0001) (Figure 4B).

When re-interrogating the GSE74685 dataset, the expression of these five factors was able to discriminate bone metastases from other metastatic sites (Figure 4C): *PAPSS2* was significantly higher in bone vs. liver and lymph node (LN), *VDR* was higher in bone vs. LN and lung, *SLC16A1* was higher in bone compared with liver, lung and LN, and *GPX1* was higher in bone vs. LN metastases (Figure 4D). *PPARA* had no significant differences (Figure 4D). When assessing the signature taking into consideration only bone metastatic samples, we observed that the hazard ratio was even higher (HR = 23.869, *p* < 0.0001) in the subgroup with high-risk score for the selected signature (Figure 4E and Supplementary Table S3B). Of note, in the SU2C-PCF dataset, patients’ survival was not dependent on the metastatic site itself (Figure 4F). Thus, these results demonstrate that transcriptomic changes of a discrete metabolic gene signature are associated with significant changes in OS, highlighting their clinical relevance for metastatic PCa.

### 3.5. Mechanism-Centric Approach to Delineate Potential Drivers of the Metabolic Rewiring of PCa Cells

**Figure 5:**
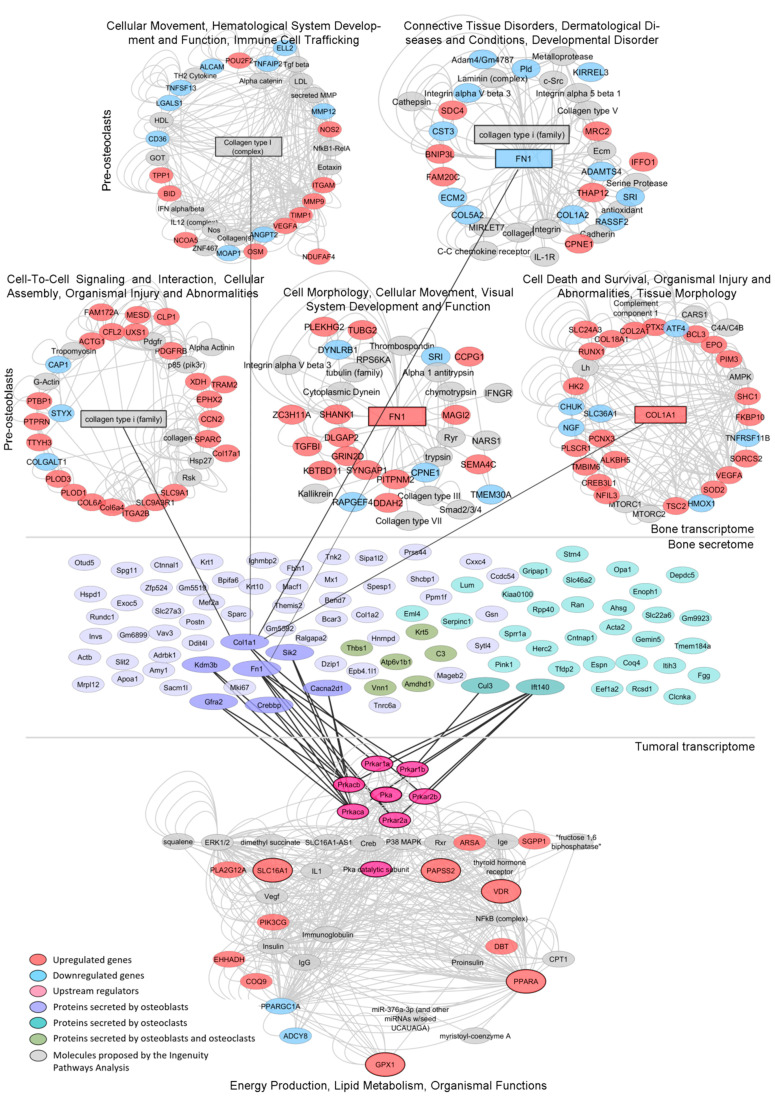
Regulation of the lipidic signature by the bone’s transcriptome and secretome. Mech-anistic model by which dysregulations in the bone cells’ transcriptome and secretome under co-culture with PC3 cells may be regulating the expression/activity of the tumor-associated PKA gene, a potential driver of the lipid-associated signature, which in turn increases the expression of *SLC16A1*, *VDR, GPX1, PPARA* and *PAPSS2*, thus shaping the metabolic phenotype of metastatic PCa. Networks from the Ingenuity Pathway Analysis (IPA) of the transcriptomes of MC3T3 (pre-osteoblasts) or Raw264.7 (pre-osteoclasts) cells co-cultured with PC3 (upper panel). Bone proteins identified by mass spectrometry of the conditioned media (CM) of MC3T3 or Raw264.7 cells co-cultured with PC3 cells (medium panel). Enriched network from the IPA of genes dysregulated in PC3 co-cultured with MC3T3 and Raw264.7 cells (lower panel). Upregulated genes (red), downregulated genes (light blue), upstream regulators (pink), proteins secreted by osteoblasts (purple), proteins secreted by osteo-clasts (light green), proteins secreted by osteoblasts and osteoclasts (green), molecules proposed by the IPA (gray).

Our results prompted us to evaluate the possible signaling mechanisms by which the expression of the lipid-associated transcriptomic signature is regulated in PC3 cells upon co-culture with bone progenitor cells. First, an IPA evidenced that *VDR*, *PPARA*, *SLC16A1*, *GPX1* and *PAPSS2* were part of the “Energy production, Lipid metabolism and Organismal Functions” network. When hierarchically ordering the network to highlight the main flow within the directed graph, Protein Kinase A (PKA) appeared as the network’s upstream regulator (Figure 5, lower panel). PKA is a cyclic AMP (cAMP)-dependent enzyme that plays several roles in the regulation of carbohydrates and lipid metabolism in response to different nutritional conditions. Thus, this enzyme emerges as a potential driver of the metabolic phenotype of PC3 cells as a consequence of the crosstalk with bone progenitors.

### 3.6. Integrative Transcriptomics and Secretomics Analyses Pin-Point a Regulatory Axis of Tumoral Metabolism Associated with the Bone Niche

Further, we aimed at identifying soluble factors secreted by bone cells that may influence the activity of tumoral PKA. We performed an in-depth proteomics analysis (LC ESI-MS/MS) of the CM of each cell line grown alone and in co-culture as previously described [17].

We identified 65 murine proteins secreted by MC3T3 cells and 38 proteins secreted by Raw264.7 cells upon co-culture with PC3 cells (Figure 5, medium panel and Supplementary Tables S4 and S5, respectively). Protein–protein interaction analyses between the secretome of each co-culture and the regulatory (RxA/B suffix) and catalytic (CA/B suffix) subunits of PKA (PRKAR1A, PRKAR1B, PRKAR2B, PRKAR2A, PRKACA and PRKACB) revealed several factors secreted by pre-osteoblasts (Col1a1, Sik2, Cacna2d1, Crebbp, Graf2, Kdm3b and Fn1) and pre-osteoclasts (Cul3 and Ift140) during the co-culture with PC3 cells that interact and/or influence PKA activity (Figure 5, medium panel). Of note, Fn1 and Col1a1 are critical for extracellular matrix organization and bone remodeling. Additionally, MC3T3 and Raw264.7 RNA-seq data showed that genes encoding these proteins were significantly upregulated by the co-culture with PC3 cells (Figure 5, upper panel). Furthermore, IPA revealed that these proteins were involved in cell-cell interactions, cell migration and tissue morphology networks (Figure 5, upper panel).

### 3.7. Validation of PKA as a Driver of the Metabolic Rewiring in PCa Cells Induced by Bone-Secreted Factors

**Figure 6:**
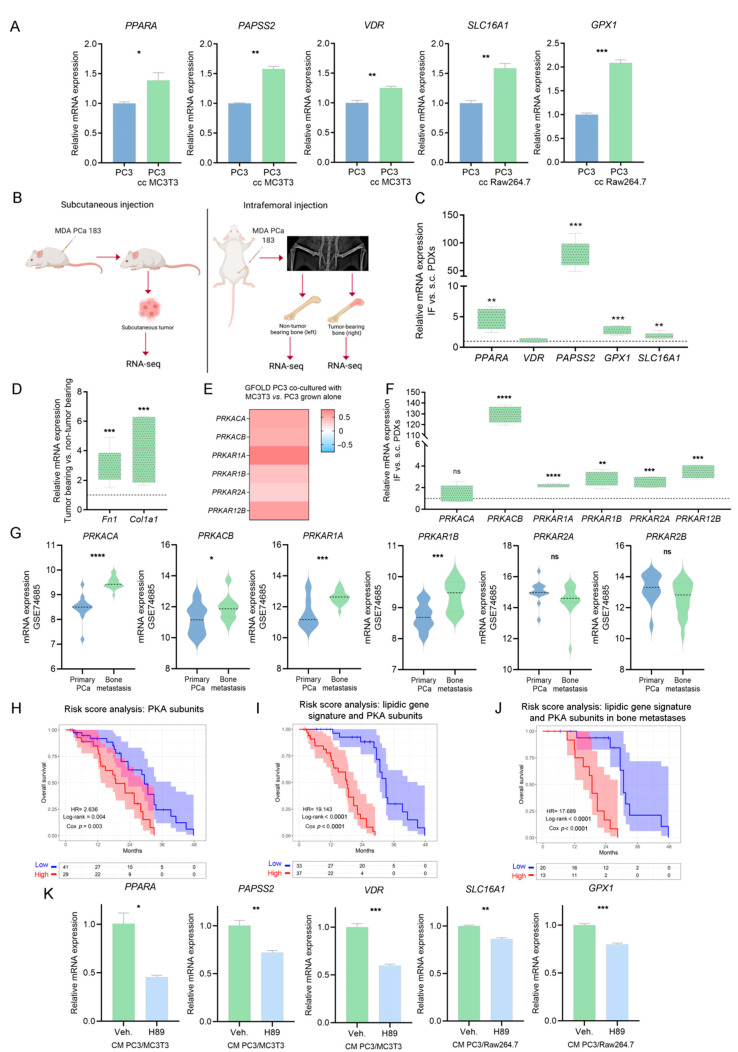
Role of PKA in the metabolic phenotype of co-cultured PC3 cells. (**A**) Gene expression validation of the lipid-associated gene signature by RT-qPCR in PC3 grown alone (PC3; control) and in PC3 co-cultured with MC3T3 or Raw264.7 cells (PC3 cc MC3T3 and PC3 cc Raw264.7, respectively). The values were normalized using *PPIA* as a reference gene and relativized to the controls. *t*-Test was used to assess statistical significance. Results are shown as the mean ± S.E.M. (**B**) Schematic representation of subcutaneous (left panel; *s.c.*) and intrafemoral (right panel; *IF*) implantation of MDA-PCa-183 PDX in male CB17 *SCID* mice and samples subsequently processed for RNA-sequencing. In the intrafemoral injection, the X-ray radiograph depicts a mouse pelvis and rear limbs at 5 weeks post-intrafemoral implantation of MDA-PCa-183-derived cells. Left legs were sham-injected non-tumor-bearing controls. (**C**) Box plots depicting IF vs. *s.c.* relative gene expression of *PPARA*, *VDR*, *PAPSS2*, *GPX1* and *SLC16A1* in MDA-PCa-183 PDX. Expression values for each gene were normalized using *PPIA* as a reference gene and *IF* was relativized to *s.c. t*-Test was used to assess statistical significance. (**D**) Box plots depicting gene expression values for *Col1a1* and *Fn1* in MDA-PCa-183 tumor-bearing vs. non-tumor-bearing bones. *t*-Test was used to assess statistical significance. Expression values for each gene were normalized using *PPIA* as a reference gene and expression in tumor-bearing bones was relativized to the expression of non-tumor-bearing bones. (**E**) Heatmap showcasing the expression (GFOLD) of PKA subunits in PC3 cells co-cultured with MC3T3 compared with PC3 grown alone. Color scale ranges from blue (GFOLD = −0.7) to white (GFOLD = 0) to red (GFOLD = 0.7). (**F**) Box plots depicting IF vs. *s.c.* relative gene expression PKA subunits in MDA-PCa-183 PDX. Expression values for each gene were normalized using *PPIA* as a reference gene and *IF* was relativized to *s.c. t*-Test was used to assess statistical significance. (**G**) Violin plots showcasing gene expression levels of the PKA subunits in bone metastasis and primary PCas from the GSE74685 dataset. Wilcoxon test was used to assess statistical significance. (**H**–**J**) KM curves for OS in high-risk (red) and low-risk (blue) groups, according to a risk score model based on the expression of PKA subunits (**H**) and PKA subunits and the lipidic gene signature (**I**) in all patients, or on the expression of PKA subunits and the lipidic genes signature in bone metastatic patients from the SU2C-PCF dataset (**J**). (**K**) Gene expression levels for *PPARA, PAPSS2, VDR, SLC16A1* and *GPX1* in PC3 cells treated with the conditioned media (CM) of the PC3/MC3T3 or PC3/Raw264.7 co-cultures, and treated with the PKA inhibitor H89 (10 µM, 3 h) or DMSO (Veh.; control). The values were normalized using *PPIA* as a reference gene and relativized to the controls. One representative of at least three independent experiments is shown. Results are shown as the mean ± S.E.M. *t*-Test’s statistical significance was set at *p* < 0.05. Statistical significance: *p* < 0.05. * *p* < 0.05, ** *p* < 0.01, *** *p* < 0.001, **** *p* < 0.0001, *ns* = not significant.

We validated by RT-qPCR the increased expression levels of *PPARA*, *PAPSS2*, *VDR*, *GPX1*, and *SLC16A1* in the co-culture systems compared to PC3 alone (Figure 6A).

Moreover, we used a pre-clinical model consisting of a PDX established from a PCa bone metastasis sample, MDA-PCa-183, grown IF or *s.c.* in CB17 *SCID* mice (Figure 6B). IF growth was monitored by X-ray (Figure 6B); *s.c.* tumors, tumor-bearing bones and non-tumor-bearing bones were isolated and subjected to RNA-seq (Figure 6B). Strikingly, IF tumors displayed increased expression levels of *PPARA*, *PAPSS2*, *SLC16A1* and *GPX1*, compared with *s.c.* tumors (Figure 6C). Expression of *Fn1* and *Col1a1* was significantly higher in tumor-bearing bones vs. non-tumor-bearing bones. These in vivo results are in line with the gene expression and secretome analyses from the in vitro co-culture experiments (Figure 6D).

Moreover, we observed a significant increase in catalytic and regulatory subunits of PKA in PC3 cells co-cultured with bone progenitors (compared with PC3 alone) (Figure 6E), in IF-grown MDA-PCa-183 PDX compared with *s.c.* tumors (Figure 6F), and in human bone metastases from the GSE74685, compared with primary PCas (Figure 6G). Additionally, a Cox regression analysis was performed to calculate a risk score including the PKA subunits showing a direct association of PKA with a worse OS in metastatic patients from the SU2C-PCF (Figure 6H and Supplementary Table S6A). Accordingly, when assessing the risk score including the lipidic gene signature and the PKA subunits, the probability of death was higher (Figure 6I and Supplementary Table S6B) and was sustained when assessed only in bone metastatic patients (Figure 6J and Supplementary Table S6C).

To decipher whether PKA exerts a regulatory function on the expression of *PPARA, PAPSS2, VDR, SLC16A1* and *GPX1*, we treated PC3 cells with the CM of each co-culture and with H89, a potent inhibitor of PKA [33]. The inhibition of PKA led to a decrease in the expression levels of these five genes (Figure 6K). Strikingly, treatment with the CM of the PC3/MC3T3 co-culture generated a decrease in the ATP content of PC3 cells compared with the control, which was restored upon PKA inhibition (Supplementary Figure S5A). The decrease in ATP levels is in accordance with the suppression of mitochondrial ATP synthesis observed in the GSEA occurring in PC3 co-cultured with MC3T3 cells (Supplementary Figure S5B), while anabolic processes such as fatty acid biosynthesis are activated (Supplementary Figure S5B), which would lead to a higher ATP consumption and a diminished ATP content. On the contrary, a significant increase in ATP content was observed upon PC3/Raw264.7 CM treatment, which was restored by the inhibition of PKA (Supplementary Figure S5C). Of note, fat digestion was activated in PC3 cells co-cultured with Raw264.7 cells (Supplementary Figure S5D), which could be the cause of a higher ATP content. These results further ascertain PKA as a potent tuner of the tumor cells’ metabolic fate upon homing in the bone niche.

This integrative analysis provided a potential signaling axis in which bone-secreted soluble factors regulate tumoral PKA, which, in turn, regulates *PPARA, VDR, SLC16A1, GPX1* and *PAPSS2* expression, leading to the metabolic rewiring of PCa cells, likely favoring disease progression.

In conclusion, these results present sound evidence that the expression profile of the lipidic gene signature represents a milestone in the metabolism of PCa bone metastases and pin-points potential druggable targets to halt disease progression.

## 4. Discussion

In this work, we identified an early metabolic rewiring towards lipid metabolism occurring in PCa cells triggered during the crosstalk with bone progenitor cells. Further, our analysis using human PCa patients’ data showcased that the lipidic gene signature found in the in vitro model, involving *PPARA*, *VDR*, *SLC16A1, GPX1* and *PAPSS2*, was enough to discriminate metastatic from primary PCa tumors, and was associated with a strong decrease in PCa patients’ survival.

Cancer cells present dysregulated cellular energetic metabolism, which is known as one of the hallmarks of cancer [40]. Lipid metabolism is one of the most altered metabolic pathways in cancer [41], as its regulation is associated with well-known oncogenic signaling axes [42], such as PI3K/Akt and Myc [43].

Despite several studies focused on primary prostate tumors and CRPC metabolism, the metabolic crosstalk within the prostate cancer-bone microenvironment remains to be elucidated. Of note, in bone metastasis there is an increase in key enzymes of the lipid biosynthetic pathway, including FASN [12,13]. Moreover, a metabolomics analysis of PCa bone metastases demonstrated significant alterations in metabolites, including an increase in the cholesterol content [44]. However, it is not clear how different cell types from the homing organ shape the metabolic phenotype of metastatic tumor cells. So far, the focus has been on adipocyte-released lipids, since it has been shown that bone metastatic PCa cells uptake FA from adipocytes in the bone marrow through the FABP4, and that this higher FA uptake may contribute to the aerobic glycolysis of PCa cells in an HIF-1α-dependent manner [9]. However, it is likely that other cells, such as osteoblasts and osteoclasts, collaborate in the metabolic reprogramming of PCa cells.

Our results show that PCa cells co-cultured with osteoblastic and osteoclastic progenitors displayed a transcriptional activation of FA metabolism, PI3K/Akt and PPARA signaling pathways. Of note, our results from PCa bone metastasis clinical samples also showed that upregulated genes were tightly associated with lipid transport, FA metabolism and other related GO categories. Thus, the co-culture model was able to mimic the transcriptional metabolic phenotype observed in bone metastatic PCa tumors.

Metabolic-targeted therapies for PCa have been focused mainly on FASN [45]. However, these therapies have failed to accomplish this goal without major side effects. Inhibiting this enzyme avoids de novo synthesis of FA, but also alters FA oxidation by inhibiting CPT1 [45], which impairs mice feeding, leading to extreme weight loss [46].

Thus, it is imperative to identify novel metabolic targets to halt PCa progression. Our research underlined the significance of lipid metabolism in PCa progression. In particular, we obtained translational results showing a responsive transcriptional signature of self-determining factors (*VDR*, *PPARA*, *SLC16A1, GPX1* and *PAPSS2)*, the alteration of which is associated with pronounced changes in OS. Additionally, understanding the upstream and downstream regulatory mechanisms of gene expression is central in the discovery of functionally relevant biomarkers and druggable targets. In this work, we propose PKA as a potential driver of the lipidic gene signature associated with the metastatic disease.

This kinase is activated by cAMP and regulates the expression of important genes during cell proliferation, migration, cytoskeleton remodeling and energetic metabolism [47]. PKA is required for cell migration on a collagen matrix as well as for Matrigel degradation by PC3M. Interestingly, our results reveal a possible extracellular regulation of PKA by bone-secreted factors, such as Col1a1. Moreover, we observed that *Col1a1* was upregulated in co-cultured MC3T3 cells; thus, bidirectional communication between tumoral and bone cells promotes a fertile niche for colonization and metastasis progression.

PKA is involved in neuroendocrine differentiation of PCa cells, leading the way to the development of resistance to Androgen Receptor (AR)-targeted therapies [48,49]. Despite AR being the most common target of current PCa treatments, progression to CRPC represents a severe clinical stage difficult to overcome. In accordance, in this work we highlight that PKA is a main driver of CRPC bone metastasis and that its mechanism involves the regulation of *VDR*, *PPARA*, *SLC16A1, GPX1* and *PAPSS2* expression. Interestingly, there are ongoing clinical trials directed to targeting these genes, such as NCT03829436 studying the anti-tumor activity of TPST-1120, a selective antagonist of PPARα, in subjects with advanced solid tumors, and a phase I/II clinical trial using AZD3965, an MCT1 inhibitor, currently being evaluated in PCa [50].

In conclusion, PKA emerges as a potential driver of the early metabolic rewiring occurring in PCa cells that interact with bone progenitors, through the expression of a gene signature highly involved in the lipidic metabolic phenotype of tumor cells. In summary, while PPARA, VDR, SLC16A1 and PAPSS2 supply the building blocks to synthesize macromolecules, GPX1 provides redox defense to counteract oxidative stress produced as a consequence of the high proliferation rates of tumor cells.

## 5. Conclusions

Overall, the integration of biological models (clinical and pre-clinical samples and the co-culture transwell system) and *omic* techniques used in this work allowed for a deeper comprehension of the dialogue between PCa cells with bone progenitors and its metabolic implications. We propose a novel communication axis where bone cells overexpress and secrete soluble factors regulating the tumoral PKA pathway, promoting the metabolic rewiring to fulfill the energetic and biosynthetic demands for bone colonization. This work highlights potential druggable targets for intervention.

## Data Availability

The data presented in this study are available on request from the corresponding author.

## Supplementary Materials

The following are available online at https://www.mdpi.com/article/10.3390/cancers14092083/s1, Supplementary Figure S1: Gene ontology analysis and unsupervised clustering analysis. Supplementary Figure S2: Expression of the metabolic genes in bone metastasis clinical samples. Supplementary Figure S3: Effect of gene expression in patients with metastatic PCa (SU2C-PCF dataset, n = 70). Supplementary Figure S4: Expression of the metabolic genes from the PC3/Raw264.7 co-culture in bone metastasis clinical samples. Supplementary Figure S5: Role of PKA in the metabolic phenotype of co-cultured PC3 cells. Table S1: Genes’ information. Table S2: Primers. Table S3: Risk score models considering the gene expression signature. Table S4: MC3T3 secretome. Table S5: Raw264.7 secretome. Table S6: Risk score models considering the gene expression signature and PKA subunits.
