# Supplementary material for: Bone Progenitors Pull the Strings on the Early Metabolic Rewiring Occurring in Prostate Cancer Cells
Source: Cancers (Basel). 2022 Apr 21;14(9):2083. doi: 10.3390/cancers14092083 (PMC9104818; doi:10.3390/cancers14092083)
Supplement: Supplementary file 1 [file cancers-14-02083-s001.zip › cancers-1692704-supplementary.pdf]

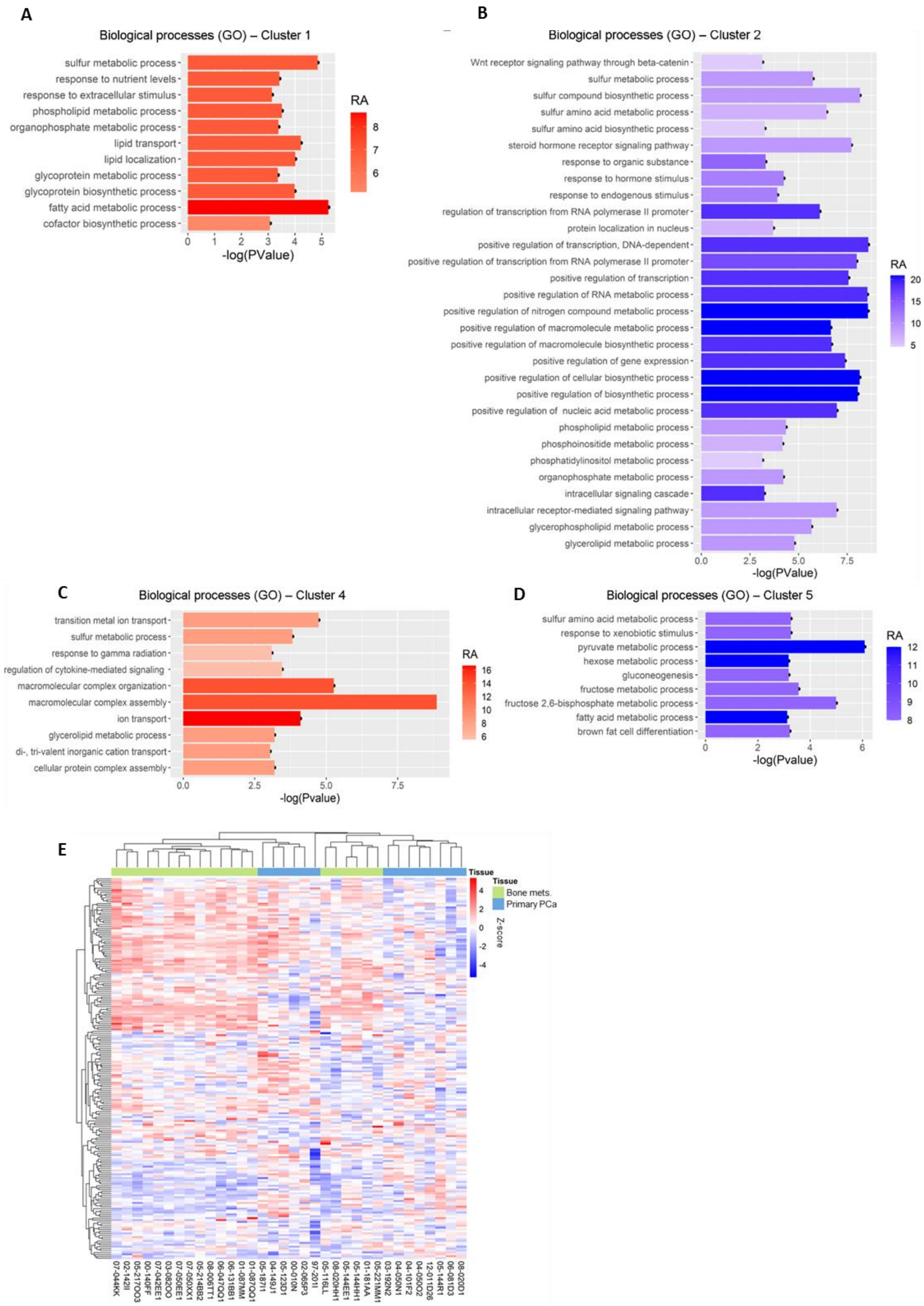

Supplementary Figure S1. Gene ontology analysis and unsupervised clustering analysis. (**A**) Bar chart of GO biological processes categories significantly enriched in Cluster 1. (**B**) Bar chart of GO biological processes categories significantly enriched in Cluster 2. (**C**) Bar chart of GO biological processes categories significantly enriched in Cluster 4. (**D**) Bar chart of GO biological processes categories significantly enriched in Cluster 5. The color intensity represents the Relative Abundance (RA) of genes included in each category. (**E**) Heatmap depicting an unsupervised clustering analysis in human primary tumor and bone metastasis samples from GSE74685, considering the expression of 200 randomly selected genes. Red, white and blue represent higher, equal or lower z-score expression levels, respectively. Each green or blue box at the top of the heatmap represents a bone metastasis or primary tumor sample, respectively.

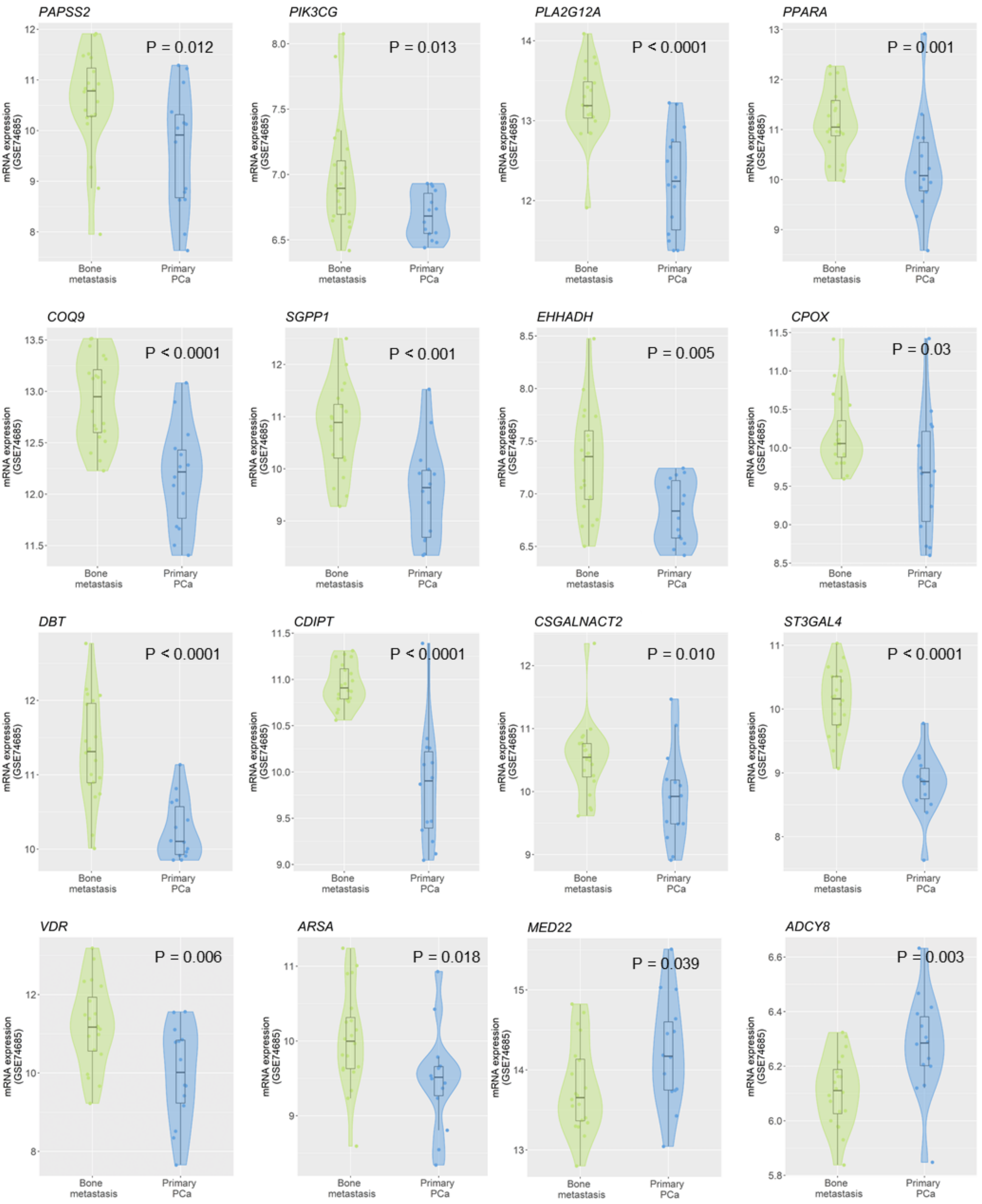

Supplementary Figure S2. Expression of the metabolic genes in bone metastasis clinical samples. Violin plots depicting gene expression levels of genes significantly dysregulated in bone metastasis compared to primary PCa samples from the GSE74685, and whose expression is also dysregulated in PC3 co-cultured with MC3T3. p values correspond to Wilcoxon rank-sum test. Statistical significance: $p < 0.05$

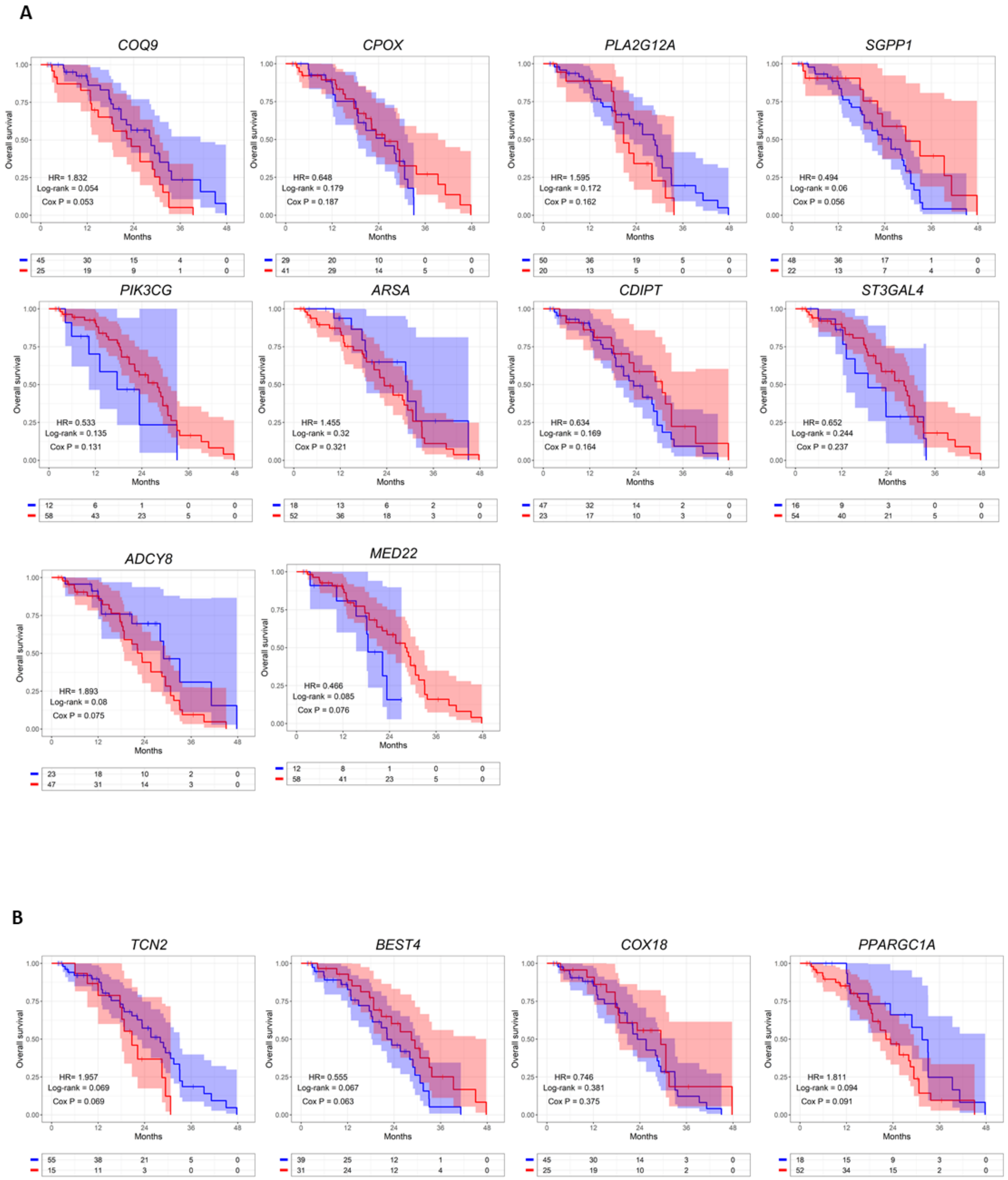

Supplementary Figure S3. Effect of gene expression in patients with metastatic PCa (SU2C-PCF dataset, n = 70). Kaplan–Meier curves for overall survival of metastatic PCa patients segregated based on the gene expression levels in two groups: high expression (red curves) and low expression (blue curves). (**A**) Genes dysregulated in bone metastasis vs. primary tumors (GSE74685) and in PC3 co-cultured with MC3T3. (**B**) Genes dysregulated in bone metastasis *vs*. primary tumors and in PC3 co-cultured with Raw264.7. All comparisons considered low expression patients as the reference group. HR: hazards ratio. Statistical significance: $p < 0.05$.

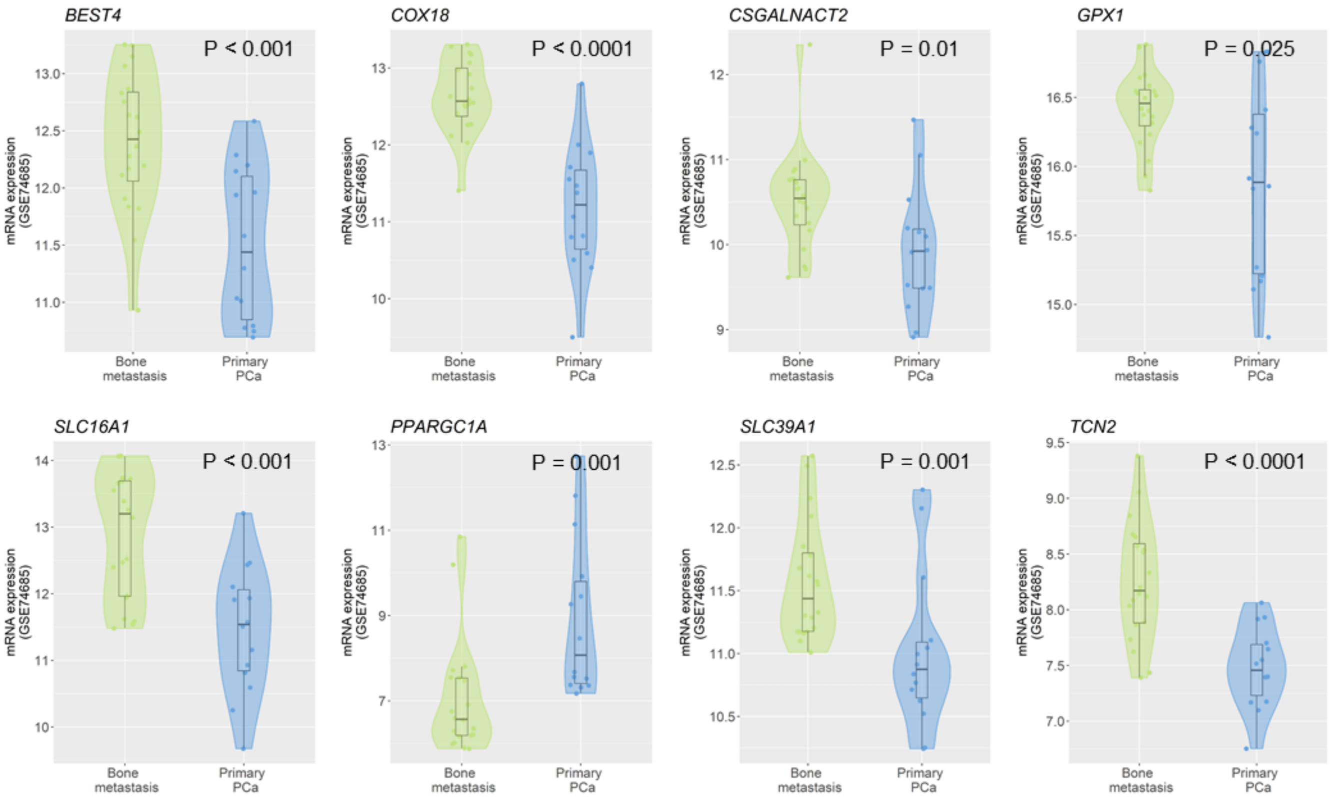

Supplementary Figure S4. Expression of the metabolic genes in bone metastasis clinical samples. Violin plots depicting gene expression levels of genes significantly dysregulated in bone metastasis compared to primary PCa samples from the GSE74685, and whose expression is also dysregulated in PC3 co-cultured with Raw264.7. *p*-values correspond to Wilcoxon rank-sum test. Statistical significance: $p < 0.05$.

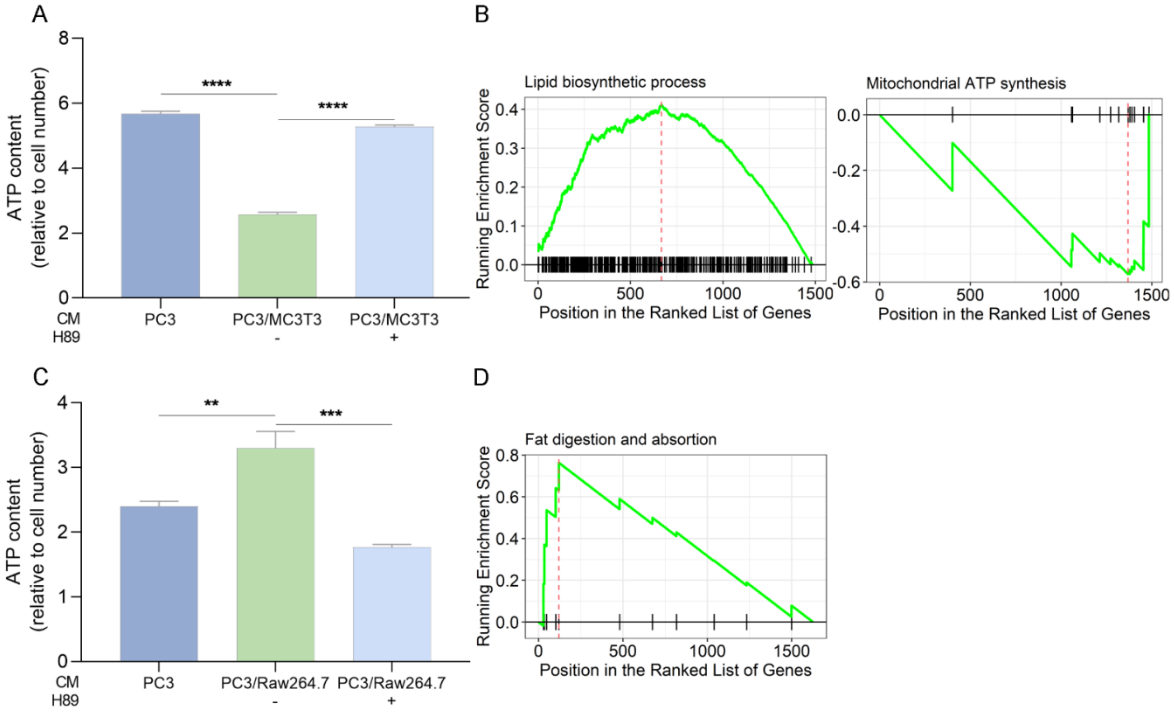

Supplementary Figure S5. Role of PKA in the metabolic phenotype of co-cultured PC3 cells. (**A**,**C**) ATP content relative to cell number in PC3 cells treated with the conditioned media (CM) of PC3 alone for 24 h, treated with the CM of the PC3/MC3T3 (**A**) or PC3/Raw264.7 (**C**) co-culture for 24 h, and treated with CM of the each co-culture for 24 h and the PKA inhibitor H89 (10 μM) in the last 3 h. *p*-values correspond to One-way ANOVA followed by Tukey test. (**B**,**D**) GSEA Enrichment plot for Mitochondrial ATP synthesis and Lipid biosynthetic process in PC3 cells co-cultured with MC3T3 compared to PC3 cells alone (**B**), and for Fat digestion and absorption in PC3 cells co-cultured with Raw264.7 compared to PC3 cells alone (D). ** $p < 0.01$; *** $p < 0.001$; **** $p < 0.0001$.

Supplementary Table S1. Genes' information. Gene Name, Gene Symbol, Entrez Gene ID and Species for every gene which expression was compared.

Gene Name	Gene symbol	Entrez_Gene ID	Species
arylsulfatase A	ARSA	410	Homo sapiens
phospholipase A2 group XIIA	PLA2G12A	81579	Homo sapiens
solute carrier family 16 member 1	SLC16A1	6566	Homo sapiens
glutathione peroxidase 1	GPX1	2876	Homo sapiens
vitamin D (1,25- dihydroxyvitamin D3) receptor	VDR	7421	Homo sapiens
COX18, cytochrome c oxidase assembly factor	COX18	285521	Homo sapiens
chondroitin sulfate N-acetylgalactosaminyltransferase 2	CSGALNACT2	55454	Homo sapiens
coproporphyrinogen oxidase	CPOX	1371	Homo sapiens
coenzyme Q9	COQ9	57017	Homo sapiens
adenylate cyclase 8	ADCY8	114	Homo sapiens
sphingosine-1-phosphate phosphatase 1	SGPP1	81537	Homo sapiens
3'-phosphoadenosine 5'-phosphosulfate synthase 2	PAPSS2	9060	Homo sapiens
phosphatidylinositol-4,5-bisphosphate 3-kinase catalytic subunit gamma	PIK3CG	5294	Homo sapiens
mediator complex subunit 22	MED22	6837	Homo sapiens
bestrophin 4	BEST4	266675	Homo sapiens
transcobalamin 2	TCN2	6948	Homo sapiens
enoyl-CoA hydratase and 3-hydroxyacyl CoA dehydrogenase	EHHADH	1962	Homo sapiens
dihydrolipoamide branched chain transacylase E2	DBT	1629	Homo sapiens
ST3 beta-galactoside alpha-2,3-sialyltransferase 4	ST3GAL4	6484	Homo sapiens
CDP-diacylglycerol--inositol 3-phosphatidyltransferase	CDIPT	10423	Homo sapiens
peroxisome proliferator activated receptor alpha	PPARA	5465	Homo sapiens
solute carrier family 39 member 1	SLC39A1	27173	Homo sapiens
PPARG coactivator 1 alpha	PPARGC1A	10891	Homo sapiens
Protein Kinase, CAMP-Dependent, Alpha Catalytic Subunit	PRKACA	5566	Homo sapiens
Protein Kinase, CAMP-Dependent, Beta Catalytic Subunit	PRKACB	5567	Homo sapiens
Protein Kinase, CAMP-Dependent, Regulatory Subunit Type I Alpha	PRKAR1A	5573	Homo sapiens
Protein Kinase, CAMP-Dependent, Regulatory Subunit Type I Beta	PRKAR1B	5575	Homo sapiens
Protein Kinase, CAMP-Dependent, Regulatory Subunit Type II Alpha	PRKAR2A	5576	Homo sapiens
Protein Kinase, CAMP-Dependent, Regulatory Subunit Type II Beta	PRKAR2B	5577	Homo sapiens
Collagen, type I alpha 1	Col1a1	12842	Mus musculus
Fibronectin 1	Fn1	14268	Mus nusculus

Supplementary Table S2. Primers. Table of primers, containing gene names, sequences (5'-3'), and annealing temperature (T° an.).

Gene	Forward primer	Reverse primer	T°an.
PPARA	AGCTGTCACCACAGTAGCTT	CAGAGTGGGCTTTCCGTGT	59 °C
VDR	GAGCCCCTCATCAAGTTCCA	AGTGTGTTGGACAGGCGG	62 °C

SLC16A1	CACCGTACAGCAACTATACG	CAATGGTCGCCTCTTGTAGA	57 °C
GPX1	ACACCCAGATGAACGAGCTG	AGCAGCTGATGCCCAAACT	60 °C
PAPSS2	CCCTGCTAGATGATGGCGTG	ACCCTTCCAGCCGTGTCTTA	56 °C
PPIA	GGTATAAAAGGGGCGGGAGG	CTGCAAACAGCTCAAAGGAGAC	60 °C

Supplementary Table S3. Risk score models considering the gene expression signature. Detailed information of metabolic genes for the risk score models for (A) all metastatic patients and (B) for bone metastatic patients from the SU2C-PCF dataset. Coef.: regression coefficient. [95% CI]: 95% confidence interval. Statistical significance: $p < 0.05$.

**Supplementary table S3A**			
**Gene**	**Coef.**	**[95% CI]**	**p**
PPARA	1.19	0.45–1.93	0.002
VDR	1.11	0.35–1.88	0.004
SLC16A1	1.56	0.73–2.39	<0.0001
GPX1	1.06	0.36–1.77	0.003
PAPSS2	−2.72	-3.86–1.58	<0.0001
**Supplementary table S3B**			
**Gene**	**Coef.**	**[95% CI]**	**p**
PPARA	1.79	0.1–3.48	0.038
VDR	−0.76	−2.38–0.85	0.355
SLC16A1	1.55	0.03–3.07	0.046
GPX1	1.55	−0.13–3.22	0.071
PAPSS2	0.29	−0.89–1.48	0.629

Supplementary Table S4. MC3T3 secretome. List of proteins for the conditioned media of PC3 cells co-cultured with MC3T3 cells identified by LC ESI-MS/MS analysis and matched against the murine protein database.

**Protein Name**	**Gene symbol**	**Entrez_Gene ID**	**Species**	**Coverage**	**# Peptides**	**# PSMs**
39S ribosomal protein L12, mitochondrial	Mrpl12	56282	Mus musculus	16.91542	1	1
60 kDa heat shock protein, mitochondrial	Hspd1	15510	Mus musculus	3.664921	1	1
Actin, cytoplasmic 1	Actb	11461	Mus musculus	21.6	5	5
Activated CDC42 kinase 1	Tnk2	51789	Mus musculus	9.311741	1	1
Alpha-amylase 1	Amy1	11722	Mus musculus	8.219178	1	1
Alpha-catulin	Ctnnal1	54366	Mus musculus	4.924761	1	2
Apolipoprotein A-I	Apoa1	11806	Mus musculus	4.166667	2	2
ATPase, H+ transporting, lysosomal V1 subunit B1	Atp6v1b1	110935	Mus musculus	6.237817	1	2
Band 4.1-like protein 1	Epb4.1l1	54357	Mus musculus	4.550626	1	1
BEN domain-containing protein 7	Bend7	209645	Mus musculus	8.294931	1	1
Beta-adrenergic receptor kinase 1	Adrbk1	156	Mus musculus	4.644412	1	1
Breast cancer anti-estrogen resistance protein 3	Bcar3	29815	Mus musculus	4.878049	1	1
Coiled-coil domain-containing protein 54	Ccdc54	69339	Mus musculus	11.2462	1	1

Collagen alpha-1(I) chain	Col1a1	12842	Mus musculus	4.40468	4	5
Collagen alpha-2(I) chain	Col1a2	12843	Mus musculus	1.093294	1	1
Complement C3	C3	12266	Mus musculus	1.683704	3	3
CREB-binding protein	Crebbp	12914	Mus musculus	1.843507	1	1
CXXC-type zinc finger protein 4	Cxxc4	319478	Mus musculus	10.10929	1	1
DNA damage-inducible transcript 4-like protein	Ddit4l	73284	Mus musculus	14.50777	1	1
DNA-binding protein SMUBP-2	Ighmbp2	20589	Mus musculus	2.719033	1	1
Exocyst complex component 5	Exoc5	105504	Mus musculus	2.966102	1	1
Fibronectin	Fn1	14268	Mus musculus	1.009285	1	1
Fibulin-1	Fbln1	14114	Mus musculus	1.702128	1	1
GDNF family receptor alpha-2	Gfra2	14586	Mus musculus	7.543103	1	1
Gelsolin	Gsn	227753	Mus musculus	2.820513	2	2
Guanine nucleotide exchange factor VAV3	Vav3	57257	Mus musculus	1.88902	1	1
Heterogeneous nuclear ribonucleoprotein D, isoform CRA_a	Hnrnpd	11991	Mus musculus	7.142857	1	1
Interferon-induced GTP-binding protein Mx1	Mx1	17857	Mus musculus	6.220096	1	1
Inversin	Invs	16348	Mus musculus	2.919021	1	1
Jumonji domain containing 1B	Kdm3b	277250	Mus musculus	1.248581	1	1
Keratin, type I cytoskeletal 10	Krt10	16661	Mus musculus	1.578947	1	1
Keratin, type II cytoskeletal 1	Krt1	16678	Mus musculus	3.767661	2	2
Keratin, type II cytoskeletal 5	Krt5	110308	Mus musculus	10.51724	2	2
Long-chain fatty acid transport protein 3	Slc27a3	26568	Mus musculus	5.845182	1	1
MCG142459	Gm5519	433241	Mus musculus	8.130081	1	1
Microtubule-actin cross-linking factor 1	Macf1	11426	Mus musculus	0.367197	1	1
Myocyte-specific enhancer factor 2A	Mef2a	17258	Mus musculus	6.626506	1	1
OTU domain-containing protein 5	Otud5	54644	Mus musculus	4.378284	1	1
Pantetheinase	Vnn1	22361	Mus musculus	2.539063	1	1
Periostin	Postn	50706	Mus musculus	2.267303	1	1
Phosphatidylinositide phosphatase SAC1	Sacm1l	83493	Mus musculus	7.495741	1	1
Predicted gene, EG545477	Bpifa6	545477	Mus musculus	8.174387	1	1
Predicted gene, EG628586	Gm6899	628586	Mus musculus	29.10448	1	1
Probable imidazolonepropionase	Amdhd1	71761	Mus musculus	11.03286	1	1

Proliferation marker protein Ki-67	Mki67	17345	Mus musculus	0.566572	1	1
Protein Mageb2	Mageb2	17146	Mus musculus	4.473684	1	1
Protein phosphatase 1F	Ppm1f	68606	Mus musculus	9.070796	1	1
Protein THEMIS2	Themis2	230787	Mus musculus	3.31825	1	1
Ral GTPase-activating protein subunit alpha-2	Ralgapa2	241694	Mus musculus	2.198953	1	1
RUN domain-containing protein 1	Rundc1	217201	Mus musculus	10.48387	1	1
Serine protease 44	Prss44	73336	Mus musculus	10.48387	1	1
Serine/threonine-protein kinase SIK2	Sik2	235344	Mus musculus	3.007519	1	1
SHC SH2 domain-binding protein 1	Shcbp1	20419	Mus musculus	5.239521	1	1
Signal-induced proliferation-associated 1-like protein 2	Sipa1l2	244668	Mus musculus	1.045296	1	1
Slit homolog 2 protein	Slit2	20563	Mus musculus	3.642773	1	1
SPARC	Sparc	20692	Mus musculus	5.298013	1	1
Spatacsin	Spg11	214585	Mus musculus	1.687243	1	1
Sperm equatorial segment protein 1	Spesp1	66712	Mus musculus	8.77193	1	1
Synaptotagmin-like protein 4	Sytl4	27359	Mus musculus	4.903418	1	1
Thrombospondin 1	Thbs1	21825	Mus musculus	0.939368	1	1
Trinucleotide repeat-containing gene 6A protein	Tnrc6a	233833	Mus musculus	1.430615	1	2
Uncharacterized protein C2orf78 homolog	Gm5592	434172	Mus musculus	3.850642	1	1
Voltage-dependent calcium channel subunit alpha-2/delta-1	Cacna2d1	12293	Mus musculus	3.44515	1	1
Zinc finger protein 524	Zfp524	66056	Mus musculus	5.607477	1	1
Zinc finger protein DZIP1	Dzip1	66573	Mus musculus	2.699531	1	1

Supplementary Table S5. Raw264.7 secretome. List of proteins for the conditioned media of PC3 cells co-cultured with Raw264.7 cells identified by LC ESI-MS/MS analysis and matched against the murine protein database.

Protein name	Gene symbol	Entrez_Gene ID	Species	Coverage	# Peptides	# PSMs
Actin, aortic smooth muscle	Acta2	11475	Mus musculus	10.87533	3	4
Alpha-2-HS-glycoprotein	Ahsg	11625	Mus musculus	2.028986	1	1
Antithrombin-III	Serpinc1	11905	Mus musculus	1.505376	1	1
ATPase, H+ transporting, lysosomal V1 subunit B1	Atp6v1b1	110935	Mus musculus	6.237817	1	1
CapZ-interacting protein	Rcsd1	226594	Mus musculus	10.43689	1	1
Chloride channel protein	Clcnka	12733	Mus musculus	4.925544	1	1
Complement C3	C3	12266	Mus musculus	0.962117	1	1
Contactin-associated protein 1	Cntnap1	53321	Mus musculus	2.454874	1	1
Cornifin-A	Sprr1a	20753	Mus musculus	25	1	1
Cullin-3	Cul3	26554	Mus musculus	5.078125	1	2
DEP domain-containing protein 5	Depdc5	277854	Mus musculus	2.011314	1	1
Dynamin-like 120 kDa protein, mitochondrial	Opa1	74143	Mus musculus	4.294479	1	1
E3 ubiquitin-protein ligase HERC2	Herc2	15204	Mus musculus	0.992556	1	1
Echinoderm microtubule-associated protein-like 4	Eml4	78798	Mus musculus	3.846154	1	1
Elongation factor 1-alpha 2	Eef1a2	13628	Mus musculus	2.37581	1	1
Enolase-phosphatase E1	Enoph1	67870	Mus musculus	16.34241	1	1
Espin	Espn	56226	Mus musculus	1.921708	1	1
Fibrinogen gamma chain	Fgg	99571	Mus musculus	2.031603	1	1
Gem-associated protein 5	Gemin5	216766	Mus musculus	0.465735	1	1
GRIP1-associated protein 1	Gripap1	54645	Mus musculus	3.464755	1	1
GTP-binding nuclear protein Ran	Ran	19384	Mus musculus	19.90741	1	1
Inter-alpha-trypsin inhibitor heavy chain H3	Itih3	16426	Mus musculus	4.16198	2	2
Keratin, type II cytoskeletal 5	Krt5	110308	Mus musculus	8.448276	1	1
Lumican	Lum	17022	Mus musculus	4.43787	1	1

Pantetheinase	Vnn1	22361	Mus musculus	2.539063	1	1
Probable imidazolonepropionase	Amdhd1	71761	Mus musculus	11.03286	1	1
Protein Gm9923	Gm9923	100040505	Mus musculus	6.20915	1	1
Protein Ift140	Ift140	106633	Mus musculus	3.07377	1	1
Protein KIAA0100	Kiaa0100	72503	Mus musculus	1.745748	1	1
Ribonuclease P protein subunit p40	Rpp40	208366	Mus musculus	9.917355	1	1
Serine/threonine-protein kinase PINK1, mitochondrial	Pink1	68943	Mus musculus	6.896552	1	1
Solute carrier family 22 member 6	Slc22a6	18399	Mus musculus	8.073394	1	1
Striatin-4	Strn4	97387	Mus musculus	16.88889	1	1
Thrombospondin 1	Thbs1	21825	Mus musculus	2.391119	2	2
Thymic stromal cotransporter protein	Slc46a2	30936	Mus musculus	8.977035	1	1
Transcription factor Dp-2	Tfdp2	211586	Mus musculus	11.39896	1	1
Transmembrane protein 184A	Tmem184a	231832	Mus musculus	9.411765	1	1
Ubiquinone biosynthesis protein COQ4 homolog, mitochondrial	Coq4	227683	Mus musculus	5.263158	1	1

Supplementary Table S6. Risk score models considering the gene expression signature and PKA subunits. Detailed information for the risk score model of (A) PKA subunits' genes and (B) PKA subunits's genes and the lipidic gene signature for all patients from the SU2C-PCF dataset, and (C) for the risk score model of PKA subunits and the lipidic gene signature in bone metastatic patients from the SU2C-PCF dataset. Coef.: regression coefficient. [95% CI]: 95% confidence interval. Statistical significance: $p < 0.05$.

**Supplementary table S6A**			
**Gene**	**Coef.**	**[95% CI]**	**p**
PRKACA	−0.64	−1.49–0.21	0.139
PRKACB	1.28	0.29–2.26	0.011
PRKAR1A	−0.52	−1.28–0.23	0.172
PRKAR1B	0.26	−0.56–1.10	0.53
PRKAR2A	0.54	−0.23–1.32	0.17
PRKAR2B	0.77	−0.10–1.64	0.082
**Supplementary table S6B**			
**Gene**	**Coef.**	**[95% CI]**	**p**
PPARA	1.56	0.52–2.60	0.003
PAPSS2	−2.09	−3.31–−0.88	0.001
VDR	1.28	0.32–2.33	0.009
SLC16A1	1.19	0.14–2.24	0.027
GPX1	1.11	0.36–1.86	0.004
PRKACA	−0.32	−1.22–0.58	0.488
PRKACB	0.84	−0.29–1.96	0.146
PRKAR1A	0.21	−0.67–1.09	0.642
PRKAR1B	0.73	−0.23–1.69	0.135
PRKAR2A	−0.13	−1.13–0.88	0.799
PRKAR2B	1.56	0.26–2.86	0.019
**Supplementary table S6C**			
**Gene**	**Coef.**	**[95% CI]**	**p**
PPARA	1.9	0.09–3.71	0.04
PAPSS2	−0.46	−2.16–1.24	0.594
VDR	1.77	−0.03–3.56	0.054
SLC16A1	0.66	−1.21–2.53	0.488
GPX1	−0.18	−2.26–1.90	0.867
PRKACA	−2.93	−4.91–−0.94	0.004
PRKACB	1.99	0.41–3.57	0.014
PRKAR1A	2.5	0.56–4.44	0.011
PRKAR1B	−2.2	−4.00–−0.39	0.017
PRKAR2A	0.56	−1.31–2.43	0.556
PRKAR2B	−1.66	−3.87–0.54	0.139
